# Open reduction and internal fixation combined with hinged elbow fixator in capitellum and trochlea fractures

**DOI:** 10.3109/17453674.2010.504612

**Published:** 2010-07-16

**Authors:** Ajay Pal Singh, Arun Pal Singh, Raju Vaishya

**Affiliations:** D-13, UCMS, Guru Teg Bahadur Hospital, Dilshad GardenNew Delhi, India

*Sir*—We read with interest the article by Giannicola et al. (2010) and commend authors for their work.

Coronal shear fractures of distal humerus have received much attention in recent years (Giannicola et al. 2010) and the consensus of treatment has been open reduction and internal fixation. We also experienced good results with these fractures after internal fixation and agree with literature that early mobilization after a stable fixation is the key to good functional results (Dubberley et al. 2006, Mighell et al. 2006, Ruchelsman et al. 2008, Singh et al. 2009, Ashwood et al. 2010).

We also agree with Giannicola et al. that a poor osteosynthesis leads to prolonged immobilization of elbow and limits the functional results. We also understand that stable fixation is sometimes difficult to achieve but most of capitellar fractures can be fixed reasonably well to allow early mobilization (Mighell et al. 2006, Ruchelsman et al. 2008, Singh et al. 2009). It seems unreasonable to supplement internal fixation in all the cases as authors have done in their series.

Supplementing stable fixations with external fixator not only increases surgical time but also subjects the patients to increased risks of complications of added surgery. We would like to ask the authors to comment on the increased cost of treatment associated with fixator used.

The high rates of reoperative procedures and the discordant results for these fractures are found in largest series (28 patients) of Dubberley et al. (2006) But these fractures were treated over a period of 10 years and by different surgeons not following a uniform protocol. In series where a single protocol was followed, the results have been excellent and reproducible (Mighell et al. 2006, Ruchelsman et al. 2008, Singh et al. 2009, Ashwood et al. 2010).

Authors report extrusion of Herbert screws occurred in 2 cases. What could be the reason for this fixation failure in spite of being supplemented by external fixator?

Though we disagree on external fixation supplementation in all types of cases, we do congratulate the authors for extending the concept of supplementation of fixation to capitellar fractures. Further studies would be required to evaluate the efficacy and laying out specific indications for this kind of supplementation.
