# Supplementary material for: Open reduction and internal fixation combined with hinged elbow fixator in capitellum and trochlea fractures
Source: Acta Orthop. 2010 Jul 16;81(4):518. doi: 10.3109/17453674.2010.504612 (PMC2917578; doi:10.3109/17453674.2010.504612)
Supplement: Related texts

# Letter: Open reduction and internal fixation combined with hinged elbow fixator in capitellum and trochlea fractures

**Published:** 2010-07-16

**Authors:** G. Giannicola, F.M. Sacchetti

**Affiliations:** Department of Orthopaedic Surgery, University “Sapienza” of Rome, Rome, Italy

*Sir*—We thank Dr. Singh for the comments.

We believe that dynamic external fixator was indicated in all patients of our series because, in each of them, an unstable elbow and/or an unstable osteosynthesis was still present after ORIF and ligaments repair. A high incidence of associated lesions was present in our series, including 4 posterior elbow dislocations, 5 medial collateral ligament tears, 4 lateral or medial epicondyle fractures and 5 comminuted fractures (Giannicola et al. 2010). Other series (Ruchelsman et al. 2008 Singh et al. 2009) reported less severe elbow injuries and a lower incidence of associated lesions compared to our series. We underlined that external fixator is not indicated in all capitulum humeri and trochlea fractures but only “in complex articular fractures, particularly in those with associated ligamentous injuries and when stable fixation of the fracture fragments cannot be obtained with ORIF”. We believe these conditions should be considered a pattern of complex elbow instability and, as that, the same therapeutic algorithm should be applied (Giannicola et al. 2010).

We agree with Dr. Singh about the increased complications related to the use of external fixator, although we reported only 1 radial neuropathy and 1 pin tract infection. Overall, our complication rate was similar, or even lower, than that reported in recent series of capitellar and throclear fractures (Ring et al. 2003, Dubberley et al. 2006). As a result, we believe that when internal osteosynthesis does not guarantee a sufficient elbow stability at the end of surgery or when severe capsule-ligaments injuries contra-indicate the early motion, it is justified to perform a concomitant dynamic stabilization with external fixator and expose the patients to the possible complications related to this procedure. In extremely difficult cases such these, the use of external fixator can improve the recovery of range of motion and possibly reduce the re-operation rate related to stiffness, instability and fixation failure.

Finally, we do not consider as fixation failure the two cases with loosened screws because such events occurred 9 and 12 months after surgery. The extrusion of Herbert screws was actually caused by bone resorption of capitellum, as a result of pseudoarthrosis in the first case and degenerative changes in the latter. Osteonecrosis, osteoarthritis and pseudoarthrosis were reported by some authors (Ring et al. 2003, Dubberley et al. 2006 Giannicola et al. 2010) while others did not report such complications (Ruchelsman et al. 2008, Singh et al. 2009). In our series, these conditions were probably related to the presence small bone fragments at the fracture site and can explain the subsequent screw loosening.

In conclusion we do not recommended a blanket supplementation in all patients with capitellum and throclea fractures but only in presence of an insufficient internal osteosynthesis and/or concomitant severe ligamentous injuries. We agree with Dr. Singh that further studies are necessary to demonstrate the efficacy of concomitant external fixation, particularly in cases with associated soft tissue injuries.

## References

[1] Ashwood N, Verma M, Hamlet M, Garlapati A, Fogg Q (2010). Transarticular shear fractures of the distal humerus. J Shoulder Elbow Surg.

[2] Dubberley JH, Faber KJ, MacDermid C, Patterson SD, King GJW (2006). Outcome after open reduction and internal fixation of capitellar and trochlear Fractures. J Bone Joint Surg (Am).

[3] Giannicola G, Sacchetti FM, Greco A, Gregori G, Postacchini F (2010). Open reduction and internal fixation combined with hinged elbow fixator in capitellum and trochlea fractures. Acta Orthop.

[4] Giannicola G, Sacchetti FM, Greco A, Cinotti G, Postacchini F (2010). Management of complex elbow instability. Chir Organi Mov (Suppl 1).

[5] Mighell MA, Harkins D, Klein D, Schneider S, Frankle M (2006). Technique for internal fixation of capitellum and lateral trochlea fractures. J Orthop Trauma.

[6] Ring D, Jupiter J, Gullotta L (2003). Articular fractures of the distal part of the humerus. J Bone Joint Surg (Am).

[7] Ruchelsman DE, Tejwani NC, Kwon YW, Egol KA (2008). Open reduction and internal fixation of capitellar fractures with headless screws. J Bone Joint Surg (Am).

[8] Singh AP, Singh AP, Vaishya R, Jain A, Gulati D (2009). Fractures of capitellum: a review of 14 cases treated by open reduction and internal fixation with Herbert screws. Int Orthop.
